# A Postdigital Perspective on Service Work: Salespeople’s Service Encounters in the Connected Store

**DOI:** 10.1007/s42438-021-00280-2

**Published:** 2021-12-27

**Authors:** Charlotte Arkenback-Sundström

**Affiliations:** Department of Applied IT, University of Gothenburg, Gothenburg, Sweden

**Keywords:** Service work, Retail salespeople, Connected service encounter, Digital technologies, Theory of practice architectures, Postdigital perspective

## Abstract

Covid-19 has disrupted global markets, accelerated the digital transformation of frontline service, and changed how service organisations, frontline service employees, and consumers interact. This article explores how digitalisation is changing retail service work from a postdigital perspective. The article draws on an ethnography of salespeople’s service encounters in speciality chain stores between July 2015 and August 2021. Using a practice theory framework (the theory of practice architectures), the article explores what conditions form salespeople’s service encounters in connected stores and how retail organisations’ digitalisation of frontline service changes salespeople’s practice of service encounters. The contributions of this article to the ongoing debate over the digitalisation of service work are twofold. On the theoretical plane, the article provides an alternative framework to labour process theory for exploring and describing service work organised around digital technologies. Secondly, it uncovers the conditions that are changing salespeople’s practice of service encounters, along with attributes associated with service work and emotional labour skills. The research shows that the connected service encounter is characterised by postdigital dialogue that involves new roles and skills in frontline service work. Overall, the findings contribute to a better understanding of how digitalisation changes action and interaction in service encounters from an employee perspective.

## Introduction

The Covid-19 pandemic has disrupted global markets; changed how service organisations, frontline service employees (FSEs), and consumers interact; catalysed the digitalisation of frontline service; and showed that FSEs’ role in the service encounter may change overnight (Faraj et al. 2021; Voorhees et al. 2020). In response to customers’ changing behaviour and government restrictions, retail businesses have expanded their online and remote service offerings and digitalised the service encounter in bricks-and-mortar stores, impacting the business and its frontline service work (service work hereafter). Crucially, before Covid-19, a shift was already underway towards self-service, artificial intelligence (AI) and robot-delivered service, transforming the respective roles of FSEs, customers, and technology in the service encounter (Bowen 2016; De Keyser et al. 2019; Huang and Rust 2021; Larivière et al. 2017). Over the past four decades, the relationship between FSEs and customers in the service encounter has been studied in service work research and service, management, and marketing research, which has generated a large and diverse range of literature (see, for example, Groth et al. 2019; Ikeler 2016; Rafaeli et al. 2017; Singh et al. 2017; Subramony et al. 2021). However, these research streams seem to have developed autonomously and with little interaction and convergence, leading to a complex and incomplete picture of FSEs’ service encounters and skills within such a rapidly changing service context.

This article responds to calls for research on the ‘forgotten frontline’ (i.e., retail, hospitality, and personal service employees) and FSEs’ changing roles as organisations digitalise their frontline service offering (De Keyser et al. 2019; Subramony et al. 2021; Voorhees et al. 2020). It explores how digitalisation changes retail service work from a postdigital perspective. While a postdigital perspective on service work certainly reflects an attempt to understand what is novel about FSEs’ relationships with the digital in service encounters, it is also about recognising how digital technologies are already embedded in and entangled with their existing service encounter practices and economic and political systems (Knox 2019).

This study draws on an ethnography of salespeople’s service encounters in speciality chain stores between July 2015 and August 2021. Using a practice theory lens known as the theory of practice architectures (Kemmis 2019; Mahon et al. 2017a, b), it explores those conditions that form salespeople’s service encounters in connected stores and how retail organisations’ digitalisation of frontline service changes salespeople’s practice of service encounters. The term ‘connected’ is used here to refer to the retail store being organised around digital service network systems to produce services, to oversee and modulate FSEs’ work, and to connect the store to other places and organisations. Adopting Autio et al.’s (2021) categorisation of digital technologies, the connected service encounter involves interaction with digital in situ and communication technologies, such as the use of mobile point of sale (mPOS) in transactions at checkout.

This article unfolds in five parts. First, the concepts of service work and the digitalisation of service encounters are introduced. Second, the framework informing the analysis — the theory of practice architectures — is elaborated. Third, the research method and key analytical process are described. The fourth section presents a summary of key findings regarding the nature of salespeople’s connected service encounters and conditions of possibility and constraint for their enactment. The final section offers conclusions around the digitalisation of service work, as well as limitations and future avenues for research.

## Service Work and the Digitalisation of Service Encounters

Service work emerged in post-industrial society in the 1970s as ‘a game between persons’ (Bell 1973: 576). In the seminal work of Surprenant and Solomon (1987: 87), the service encounter was defined as ‘the dyadic interaction between a customer and a service provider’. In service research, the service encounter has been primarily regarded as a game of people driven by specific learned behaviours relevant to the situation (i.e., roles) and formulated in the organisation’s service script, a detailed guide for FSEs to follow during a service encounter. Since marketing shifted its theoretical focus from a provider to a customer perspective on customer value creation in around 2000, marketing theory and research have increasingly moved beyond the service encounter as a dyadic interaction between a firm and a customer. In the service encounter as a service ecosystem, FSEs and customers are human actors in service systems structured around digital technologies, multi-actor service systems, and networks with multiple service providers (Bowen 2016).

The service work research is characterised by a longstanding debate about service work that expands on the seminal work of Hochschild (1983) and Leidner (1993), who introduced the concepts of emotional labour and the trilateral relationship between employer, FSE and customer (the service triangle). Emotional labour describes what service workers do that goes beyond their physical or cognitive duties; it is the process by which FSEs conjure or repress specific emotions in the services they provide to others by following the service script, thereby adding value to their service or labour (Webb 2012). Much of the service work research draws on labour process theory and has focused on emotions and their management by workers (Bolton 2004), workers’ interaction with customers (Korczynski 2002), and emotional labour skills (Nickson et al. 2003; Payne 2009; Warhurst et al. 2012). Emotional labour skills are commonly explored in terms of the dynamics of gender, power, and status and not as a phenomenon in itself (Wharton 2009). These skills are also considered in relation to automation (Ilsøe 2017). For instance, in an empirical study of frontline sales work in US department stores, Ikeler (2016) found that the automation of frontline service is connected to a downgrading of salespeople’s emotional labour. However, the impact of digitalisation on emotional labour and FSEs’ service encounters has been neglected in emotional labour research.

Over the past two centuries, recurrent warnings have been raised concerning how new technology and automation will wipe out low- and middle-class jobs and transform people’s working lives. With the introduction of concepts like a ‘second machine age’ (McAfee and Brynjolfsson 2014), the discussion on potential automation of human labour, technological unemployment, and the future of work and education has intensified (Frey and Osborne 2017; Sellar 2019; Spencer 2018; Wilson 2019). While Frey and Osborne (2017) prophesy that sales occupations such as sales assistant, cashier, teller and clerk will soon be subject to a wave of computerisation, Sellar (2019) contends that since those routine tasks that are susceptible to automation are often difficult to separate from non-routine tasks that require interpersonal interaction, flexibility, adaptability, and problem solving, anxieties about automation are often overstated. Another argument advanced in the discussion is that service work is essentially human and can never be substituted by AI or robots (Frude 2019). Ikeler (2016), on the other hand, argues that emotional labour can follow a trajectory related to that which has been long apparent in manufacturing and clerical work.

We have already seen how computers, teller machines, and self-scanning checkouts are increasingly replacing cashiers and clerks in supermarkets, department stores, and banks (Basker 2016). In recent years, the intense development of big data systems, smartphone technologies, mobile tracking devices, near field communication (NFC) technology, and AI has increased the level of electronic control and surveillance of FSEs’ activities (Evans and Kitchin 2018). As a result, service organisations’ use of such technologies has severe implications for entry-level positions like cashiers and sales assistants (Barocas and Levy 2016), as well as home-care workers (Moore and Hayes 2017) in terms of job security, work schedules, financial well-being, and skills development opportunities. This study focuses on salesperson positions in speciality stores, which are primarily held by women with limited training opportunities, low wages, and insecure part-time employments (Howcroft and Rubery 2019; Tolich and Briar 1999; Webster 2004). Voorhees et al. (2020) and Larivière et al. (2017) have outlined several issues that need to be addressed in service research concerns, including how FSEs accept their new roles, how training and education can help avoid employee/customer resistance to change, and how companies can help adapt and train employees and customers to their new roles in the service encounter.

## Framework—the Theory of Practice Architectures

The theory of practice architectures (TPA), first articulated by Kemmis and Grootenboer (2008), is an account of what practices are composed of (e.g., instructing, selling, purchasing) and how they both shape and are shaped by the conditions (referred to as arrangements) that exist in, are brought to, or are newly created in a site of practice. Importantly, a site can be a physical site, such as a shopfloor or store, or a site in space and time, such as the site of a daily coffee break (Mahon et al. 2020, 2017a, b). This article follows Kemmis (2019) in defining practice as:

A form of human action in history, in which particular activities (doings) are comprehensible in terms of particular ideas and talk (sayings), and when the people involved are distributed in particular kinds of relationships (relatings), and when this combination of sayings, doings and relatings ‘hangs together’ in the project of the practice (the ends and purposes that motivate the practice). (Kemmis 2019: 13)

**Fig. 1:**
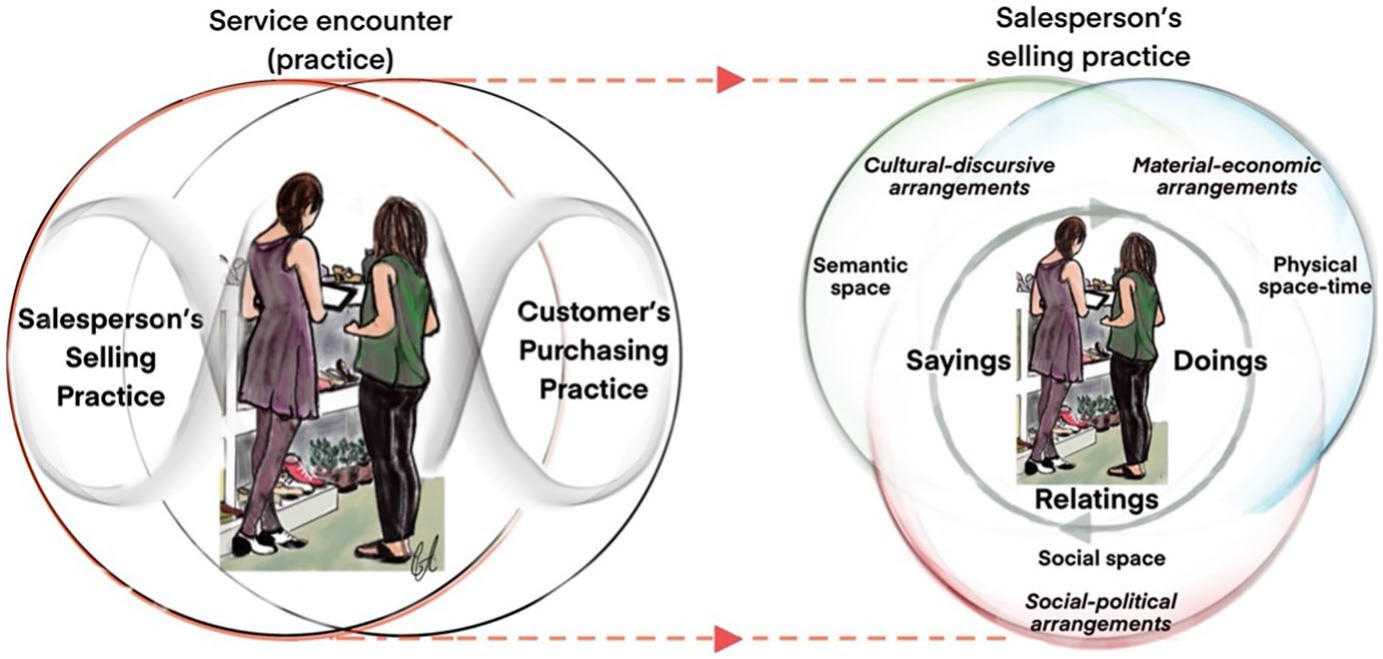
(left) Service encounter co-produced by salesperson’s selling and customer’s purchasing. (right) The salesperson plays her role through the sayings, doings, and relatings that constitute her selling practice (Kemmis et al. 2014: 33)

This article theorises the service encounter as a service practice occurring at a distinct moment when a salesperson and customer interact on the shopfloor, at checkout, or online. Such service practices are understood to be co-produced by the salesperson’s selling practice and the customer’s purchasing practice; selling and purchasing are ecologically interdependent (see Fig. 1). This idea is informed by Kemmis et al.’s (2020) conceptualisation of teaching and learning as ecologically connected in a pedagogical practice. (To facilitate reading, the service practice will hereafter be referred to as the service encounter.) As indicated in Kemmis’ (2019) aforementioned definition of practices, according to TPA, practices are composed of sayings, doings, and relatings that are interconnected in the project or the purpose of a practice. In the service encounter, the customer plays her role through the sayings, doings, and relatings that constitute her purchasing practice, and the salesperson plays the role of responding to the customer’s practice through her own sayings, doings, and relatings that form her selling practice. In this article, the analysis is focused on salespeople’s selling practices; the customer’s purchasing practice is viewed as part of the conditions that enable and constrain salespeople’s actions and interactions in the selling practice.

In TPA, practices are not seen to be directed merely by the intentions, skills, and capabilities of those who enact them; they are also steered more generally by and among conditions — cultural-discursive, material-economic, and social-political arrangements — that exist in, are brought to, or are newly created in a site of practice. In combination, these arrangements form the practice architectures that make practices possible. In turn, the practice architectures are what enable and constrain our actions and interactions, and thus the way the practice unfolds. According to TPA, the practices and the arrangements that steer them are entangled in a three-dimensional intersubjective space in which we encounter one another in semantic space, physical space–time, and social space (Kemmis 2019; Mahon et al. 2020). The key concepts of TPA also constitute a framework for analysing service encounters and prompt certain kinds of questions. In Fig. 1, the model on the right shows an abstraction of the salesperson’s selling practice.

In the dimension of semantic space, the salesperson and cashier encounter one another as interlocutors in the medium of language. They can be seen to employ cultural-discursive arrangements (for instance, the language used and issues discussed) that prefigure what is said. These cultural-discursive arrangements enable and constrain what is said (sayings) and are evident in the salesperson’s cognitive understandings of the service encounter and selling.

In the dimension of physical space–time, the pair encounters one another and the world as embodied objects in the medium of activity and work. They do this by employing material-economic arrangements: for instance, items, digital devices, and methods. The material-economic arrangements enable and constrain what is done (doings) in the selling practice and are evident in the salesperson’s skills and capacities.

In the dimension of social space, the salesperson and customer encounter one another as social beings. Social-political arrangements are realised in relation to issues of solidarity and power. For instance, the employer’s service script prefigures but does not predetermine. Therefore, the social-political arrangements enable and constrain the relationships (relatings) evolving in the practice and are evident in the salesperson’s values, feelings, and emotions (cf. Korczynski 2005; Nickson et al. 2003; Warhurst et al. 2012).

According to TPA, the sayings, doings, and relatings of practices are bundled together in the projects (purposes) of selling practices and in the salesperson’s situated knowledge about their role in the service encounter. The arrangements of the salesperson’s selling practices are bundled together in practice landscapes with other practices inside and outside the store (e.g., managers’ and stakeholders’ practices). Likewise, practice architectures are bundled together in the practice traditions that reproduce (sometimes with modifications) a practice over time as action in history (Kemmis 2019; Mahon et al. 2017a, b).

## Method

The study of salespeople’s service encounters in connected speciality chain stores was conducted through the ethnographic methods of participant observation, field interviews, field notes, and journaling between July 2015 and August 2021 (Czarniawska 2007; O'Reilly 2012). Online research was employed to gather data about retail chain organisations (Webb 2017). The workplaces included in the study were selected through convenience and purposive sampling based on the following criteria: (1) online presence (e.g., website, online store), (2) use of social media marketing, (3) status as part of a retail chain, and (4) use of mPOS systems for transactions. The final sample comprised 11 retail chain organisations (spanning fashion, apparel, intimate apparel, shoes, beauty, hair, and eyewear), 21 workplaces, and 36 participants (35 saleswomen, 1 salesman). The retail chain organisations, stores, and participants were all assigned pseudonyms. Nine of the retail organisations used online commerce, and all but one workplace was situated in Sweden (Table 1).

**Table 1:** Overview of retail chain organisations, online commerce, workplaces, participants, research methods, and timeline

**Retail chain organisations** (as of December 2020)	**Online store**	**Workplaces**	**Participants**	**Methods**	**Period**
**The Anderson organisation (Intl.)** Fashion chain 369 bricks-and-mortar stores	1998	*1 Anderson store (USA)*	1 salesperson	1 overt participant observation	2015
**The Bengtson organisation (Intl.)** Apparel chain 4 739 bricks-and-mortar stores	1998	*1 Bengtson store (SE)*	2 salespersons	4 covert and 1 overt participant observation, 1 field interview	2019–2021
**The Carlson organisation (Ntl.)** Apparel chain 90 bricks-and-mortar stores	2010	*4 Carlson stores (SE)*	1 store manager, 3 salespersons	6 covert and 4 overt participant observations, 9 field interviews	2015–2021
**The Davidson organisation (Ntl.)** Apparel chain 50 bricks-and-mortar stores	2013	*2 Davidson stores (SE)*	3 salespersons	3 covert and 3 overt participant observations, 2 field interviews	2019
**The Ericson organisation (Intl.)** Intimate apparel chain 55 bricks-and-mortar stores	2010	*2 Ericson stores (SE)*	1 store manager, 2 salespersons	3 covert and 2 overt participant observations, 1 field interview	2019—2021
**The Fredrikson organisation (Ntl.)** Shoe chain 9 bricks-and-mortar stores	2018	*2 Fredrikson stores (SE)*	2 store managers, 3 salespersons	1 covert and 4 overt participant observations, 1 work shadowing, 10 field interviews	2015–2019
**The Gustafson organisation (Intl.)** Beauty chain 250 bricks-and-mortar stores	2010	*3 Gustafson stores (SE)*	6 salespersons	6 covert and 9 overt participant observations, 5 field interviews	2015–2021
**The Hanson organisation (Intl.)** Beauty chain 730 bricks-and-mortar stores	2013	*1 Hanson store (SE)*	3 salespersons	5 covert and 6 overt participant observations, 5 field interviews	2019–2021
**The Isakson organisation (Intl.)** Jewellery and accessory chain 165 bricks-and-mortar stores	2016	*2 Isakson stores (SE)*	1 store manager, 2 salespersons	1 covert and 1 overt participant observations, 2 work shadowing, 3 field interviews	2015–2018
**The Janson organisation (Ntl.)** Hair chain 8 bricks-and-mortar stores	No	*1 Janson store (SE)*	1 store manager, 1 salesperson	1 covert and 2 overt participant observations, 3 field interviews	2019–2021
**The Knutson organisation (Ntl.)** Optician and eyewear chain with 66 bricks-and-mortar stores	No	*2 Knutson stores (SE)*	4 salespersons	5 covert and 4 overt participant observations, 3 field interviews	2018–2021
**11 retail chains**		**21 workplaces**	**36 participants** (35 saleswomen, 1 salesman)	**35 covert observations (7 h), 37 overt participant observations (12 h), 3 work shadowing (3 h), 42 field interviews**	**July 2015–August 2021**

As customers, one can relate to retail service encounters as well as certain preconceptions about salespeople’s work and skills. Ethnographic participant observation (overt, covert, shadowing) is the only field method that allows the researcher to transcend these notions and detect the realities of salespeople’s role in the service encounter. In this study, covert participant observations involved observing (from a distance) a salesperson interacting with a customer on the shopfloor and at checkout; alternatively, I would interact with the salesperson in a customer role without revealing my identity.

While the covert observations provided rich contextualised data, the field interviews, overt participant observations, and work shadowing yielded more detailed findings. In the overt observations, I engaged in a service encounter on the shopfloor, aiming to purchase an item. I then presented myself and the research before entering a dialogue about the various parts of the selling practice, ending at the checkout. The field interviews had to be short, as they were frequently interrupted by customers in need of assistance. I photographed or video recorded the store’s interior, in situ technologies, and general scenarios without showing people’s faces. This complemented the field notes written outside the store, as well as online research to gather data about the retail chain companies’ business model, digitalisation process, technological collaborations, and in-store activities. In total, the data selected for detailed analysis comprised 42 field notes, three interview transcripts, 30 photos, eight video recordings, journals, documents, and five online videos.

### Analysis Process

The analysis of the empirical data involved various processes and layers and was approached flexibly and iteratively between 2015 and 2021. Some of these processes required the continuous production of field notes, photos, journaling and journal analysis, transcript preparation, and the use of TPA and its concepts to create diagrams and maps and to write narratives about salespeople’s selling practices. TPA prompted certain kinds of questions that were helpful in the interpretive process and guided the analysis on an implicit level (Mahon et al. 2017a, b). However, they were also embedded more systematically and explicitly in specific tools developed to aid the analysis, including an analysis template based on TPA to address the particularity of connected service encounters.

Typically, the analysis process was initiated by using the key concepts of *sayings*, *doings*, and *relatings* as rubrics to organise the data material. This step was guided by analytical questions prompted by TPA, for instance: What are salespeople saying and doing in service encounters? How do they relate to digital technologies in service encounters? Following this, the conceptual rubrics, *cultural-discursive*, *material-economic*, and *social-political arrangements* were used in a similar way, for example: What arrangements specifically enable and constrain selling practices on the shopfloor and at checkout? The findings and data material were then used to produce relevant narratives, maps, and diagrams.

One issue to be handled when using TPA as a framework is how to recognise different elements as one kind of arrangement or another when they are so closely intertwined in reality. As the research evolved, my knowledge about the workplaces, selling practices, and the retail chain organisations’ digitalisation process increased. At the end of the research, I compared the narratives and findings, shifted elements to other kinds of arrangements, and searched for evidence on how new conditions (e.g., self-checkouts, omnichannel, Covid-19) impact selling practices (resist, adapt, transform) in different workplaces. Five of the stores closed between January 2020 and August 2021; for three other stores, the ownership of the retail chain organisation changed.

## Findings

The analysis of the selling practices — the sayings, doings, and relatings held together in the project of a practice — of the participants in the study yielded insights into the nature of salespeople’s selling practices and how retail organisations’ digitalisation of frontline service affects emotional labour and customer service in the service encounter. In the following sections, a summary of conditions that form the practice architectures of selling practices is presented, and some of the key conditions identified as creating tensions are described and discussed.

### Conditions Forming the Practice Architectures of Salespeople’s Selling Practices

A postdigital perspective posits that the digital is no longer new, and disruption is now mundane (Knox 2019). Indeed, this holds true for salespeople’s selling practices, where checkout transactions were first digitised in the 1970s. In this research study, salespeople worked in connected stores, leveraging interactive mPOS and service network systems.

**Fig. 2:**
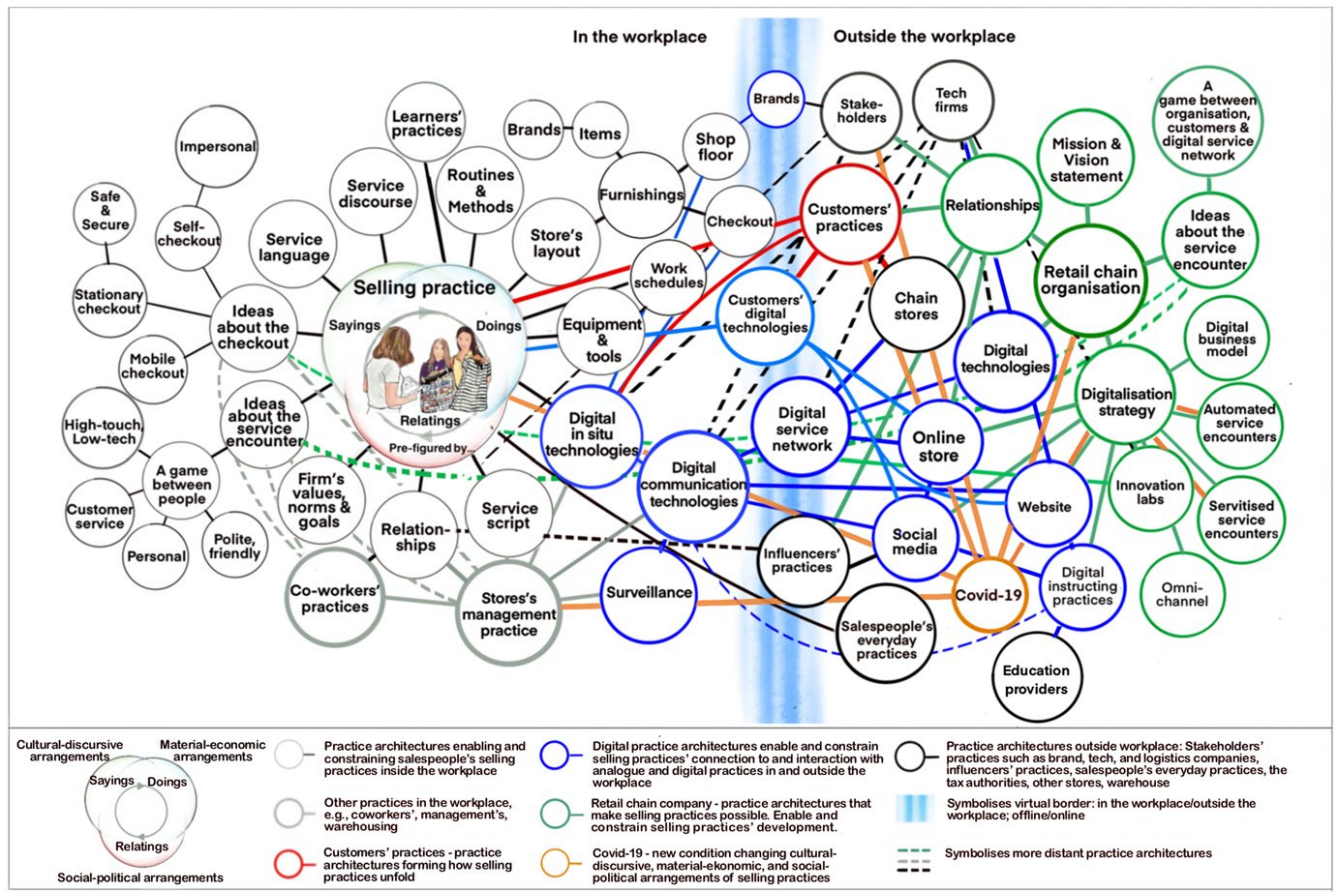
Web of conditions forming the changing practice architectures of salespeople’s selling practice part of the connected service encounter in speciality stores (as of August 2021)

The key findings of the research regarding conditions that enable a selling practice as part of the connected service encounter are synthesised in Fig. 2. On the left side of the diagram are the conditions — represented as cultural-discursive, material-economic, and social-political arrangements — that make its enactment in the workplace possible. However, in reality, these arrangements are inseparable in the salesperson’s actions and interactions (sayings, doings, relatings) in the selling practice. The right side of the diagram shows conditions and practices outside the workplace that impact how selling practices unfold and transform. For instance, in several of the stores, brand stakeholders were present on the shopfloor via QR codes and video walls. By scanning the QR code with a mobile sales assistant (MSA) or smartphone, the salesperson and customer could, for instance, test virtual products. The blue circles and lines demonstrate how salespeople and customers are human actors in the retail chain organisation’s service systems, which are structured around digital technologies, multi-actor service systems, and networks with multiple service providers (Bowen 2016; De Keyser et al. 2019; Larivière et al. 2017). The blue vertical lines symbolise the virtual border of selling practices, connecting them (and the store) in real time to other practices and locations, both online and offline.

However, while offering a model for visualising conditions that enable and constrain what could be defined as ‘postdigital selling practices’, the diagram is also complex and dense with information. Depending on the context and service work knowledge, this could give rise to different interpretations and aspects of the digitalisation of retail service work. Knox (2019) suggests that the ‘postdigital might be understood as a kind of ‘holding to account’ of many of the assumptions associated with digital technologies in education’ (2019: 368). Many such assumptions are associated with emotional labour, including salespeople, sales assistants, and cashiers in retail (Ikeler 2016; Nickson et al. 2012), an industry that has been particularly characterised by digital transformation (Hagberg et al. 2017; Yrjölä et al. 2018). Nonetheless, accepting that salespeople’s connected service encounters can be discussed in terms of practices made possible by particular conditions and that these conditions can analytically be represented in Fig. 2; then, the diagram can serve as a ‘map’ to explore and discuss conditions and circumstances that make salespeople resist, adapt, or approach their practice differently in the connected service encounter. Hence, the following sections will unpack and comment on certain conditions from Fig. 2 that provoked tensions in the salespeople’s part of the service encounter.

### Digital Technologies

Technological advancements have profoundly impacted how companies organise their frontline service. Increasingly, digital technologies — analytically conceptualised as material-economic arrangements — have supplemented or substituted FSEs as the service provider (Brynjolfsson and McAfee 2011). Here, the analysis revealed that four of the retail chain organisations had collaborated with technology firms on smartphone, AI, VR, and AR innovations to create ‘extraordinary customer experiences’ and enhance the company-customer relationship. For instance, Anderson reported developing digital in situ and communication technologies for service encounters in collaboration with salespeople and customers in innovation labs. In 2015, a number of stores installed smart mirrors in their fitting rooms, which suggest outfits to go with the garment that the customer has chosen to try on. Another example is Hanson, who in 2017 used VR headsets and a 360° video to immerse customers in an authentic hammam setting in flagship stores around the world and online via social media. These findings corroborate a great deal of the marketing and service literature discussing the digital transformation of service encounters (Brynjolfsson et al. 2013; De Keyser et al. 2019; Verhoef et al. 2015). Thus, salespeople’s service encounters in the connected speciality chain stores could be expected to be both entangled in and constituted by pervasive digital technologies.

**Fig. 3:**
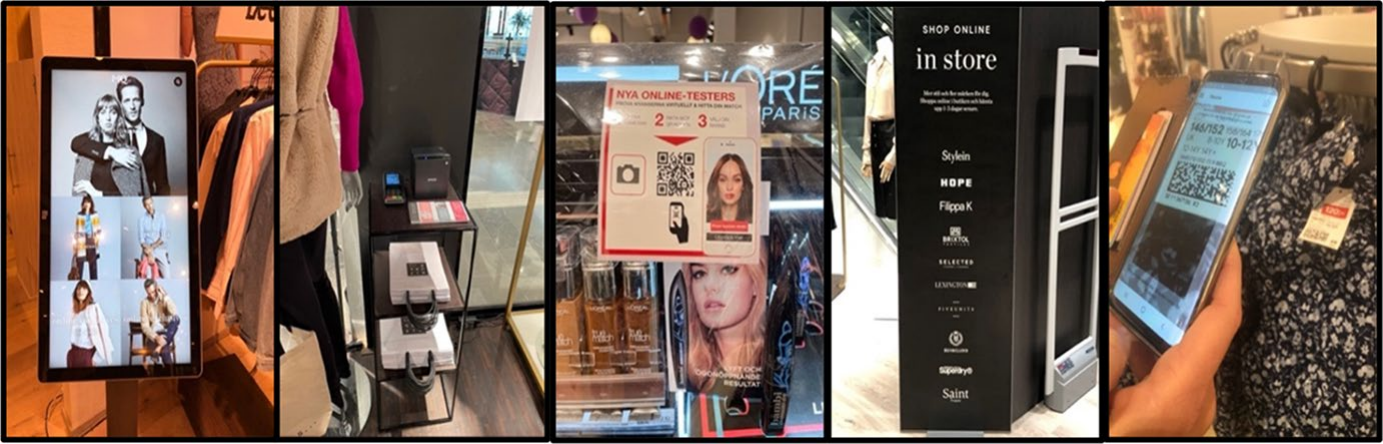
Touchpad, self-checkout, cosmetic brand QR system, and smartphone application to shop online in-store

New digital in situ and communication technologies do not appear in isolation in work practices. They come with other arrangements, such as new routines, methods, organisational goals, and values. Nevertheless, signs of technology enhancing salespeople’s emotional work in service encounters were scarce. Similarly, the introduction of touchpads, QR systems, and MSAs did not involve any observable changes in the stores’ layout or decor (Fig. 3).

Analogue signage on the shopfloor informed the intertwining of the physical and online stores. A touchpad on the shop floor provided access to the online store in five of the stores surveyed, though the salespeople’s online activities were typically performed at the fixed checkout. One key finding is that the digital in situ technology varied between stores within the same retail chain. As Erica notes from her work at one of the Ericson stores:

We do not have a touchpad. Our store is not that modern, but I think the store downtown has one. If the customers want to shop online or collect online purchases, they must approach us at the checkout. (Erica at Ericson February 2021)

This is just one example of how digital technologies and services — or the lack thereof — shape the enactment of service encounters and salespeople’s values and relationship to the workplace. Most participants perceived frontline innovations such as smart mirrors or AR and VR technologies as fiction. From their point of view, such technologies were unlikely to be introduced in their workplaces for the foreseeable future.

Furthermore, it emerged that the use of smartphones, social media, online shopping outside work, and positive relationships with digital technology were not sufficient conditions for the participants to adopt in situ technologies such as MSAs in service encounters on the shopfloor (cf. Spreer and Rauschnabel 2016).

### Ideas on the Service Encounter

The contrasting understandings of salespeople and retail chain organisations — analytically conceptualised as cultural-discursive arrangements — showed a condition constraining salespeople’s actions and interactions in the connected service encounter. Here, the traditional conceptualisation of the service encounter as a game between people (cf. Solomon et al. 1985) with little involvement of digital technologies has shaped how service encounters evolved. The participants uniformly described the motivation for working as a salesperson as the personal interaction with customers and the possibility of making them feel valuable and satisfied. On the other hand, the retail chain organisations understood the service encounter as a game between the organisation, customers, and digital service network, whereby the border between digital and physical service encounters is blurred (cf. De Keyser et al. 2019; Larivière et al. 2017). The nine retail organisations with online stores reported transformation to omnichannel commerce, analytically categorised as a social-political arrangement that changes salespeople’s role in the service encounter. Omnichannel refers to blending speciality bricks-and-mortar stores with online commerce to create a seamless customer shopping experience with multiple touchpoints (Brynjolfsson et al. 2013; Verhoef et al. 2015). During the research, the omnichannel strategy became more pronounced in the individual stores part of retail chain organisations with online stores.

It is well known that changing established practices can be difficult (Kemmis 2019; Shove et al. 2012). Thus, what happened in practice when digital technologies connecting the speciality stores with digital services and the online store were introduced? First, digital in situ technologies were perceived by all the participants as intended to enhance customers’ purchasing practices. As noted by the store manager at Davidson:

The touchpad is valuable. We have it for the customers, so they do not have to wait for help if there is a queue at checkout or the staff are occupied elsewhere (Manager at Davidson February 2019).

Second, within a year, the touchpads on the shopfloor had been removed in the stores. Asking why they had disappeared, one participant explained:

The touchpad? Yes, that is right. I remember we had one for a while, but no customers used it, and we have everything we need at the checkout. It was mainly in the way on the shopfloor, so they took it away. (Celia at Carlson January 2020)

These comments illustrate how digital in situ technologies, in the form of mPOS devices, are located at the fixed checkout in salespeople’s service encounters. What could be seen was that salespeople’s connected service encounters continued as a game between people, starting on the shopfloor and ending at the checkout. No evidence was found of employers’ service scripts for service encounters changing as the retail chain organisations digitalised service encounters.

### The Checkout

The term ‘checkout’ had various meanings in salespeople’s practice of service encounters. First, it referred to the in situ technologies constituting the mPOS system (i.e., PC register, cash box, screen, scanner, credit card reader, keyboard, mouse, receipt printer, mPOS software). Second, it referred to a site in the store with a particular layout and furnishings (e.g., checkout counter, mPOS system, mobile devices, shelves, tools, bags). Third, it referred to the service encounter at checkout, the checkout practice, which comprises two parts: *transactions* and *customer service*. The phrase ‘go to checkout’, meanwhile, signalled activities in the service encounter that were not performed on the shopfloor; these included searching for information, handling situations (e.g., angry customers, refunds), problem-solving (e.g., supporting online purchases), and online ordering. In summary, checkout is not merely a site for transactions that can be automated (cf. Brynjolfsson and McAfee 2011; Frey and Osborne 2017); it is the salespeople’s office on the shopfloor in which excellent customer service is created.

While salespeople’s service encounters on the shopfloor were primarily a game between the salesperson and customer, the service encounter in the checkout office was also a game between the salesperson, customer, and service network systems. In the checkout practice, the salesperson engaged in digital dialogue with the mPOS (connected to transactions) in parallel with organic dialogue with the customer (connected to customer service). The more retail management systems intertwined with the mPOS, the more emphasis was placed on the mPOS dialogue. The salesperson responded to prompts on the screen and produced and mediated information between the customer and the mPOS. It was found that closing a sale of two items involved interacting with between 12 and 25 windows on the screen. On the other hand, the customer dialogue was shaped by a service discourse and involved a combination of information-giving and small talk: for example, ‘It is a good choice, I have bought one myself and I love it’.

Jandrić et al. argue that ‘there is no such thing as ‘purely digital’ dialogue or ‘purely analogue’ […] today’s dialogue is inherently postdigital’(Jandrić et al. 2019: 164). Introducing the concept of postdigital dialogue is useful for visualising the points of difference between traditional and connected service encounters that are essential to understanding the evolution of service work. For a long time, retail service work has been considered as positions requiring little or no qualifications other than interpersonal and customer service skills (Hastwell et al. 2013; Nickson et al. 2012). However, participating in a postdigital dialogue in the connected service encounter required numeracy, literacy, and digital skills in combination with emotional labour (Bélanger and Edwards 2013; Groth et al. 2019).

In most of the stores analysed, separate digital in situ technologies were employed to handle in-store and online purchases. On the one hand, the mPOS systems were perceived as easy to operate. On the other hand, the participants hoped the company would not change to a new system, which would take a long time to learn. One example of how the digital transformation of frontline service increases the complexity of salespeople’s connected service encounters was observed in one of the Knutson stores. Starting in 2018, in my first three meetings with Karin, an experienced salesperson, she was able to handle the mPOS system without difficulties. However, in 2021, the purchase of sports eyewear was proving problematic:

I am sorry it takes time. It is not our brand. Here it is... No, that must be wrong, 1,500, you should not pay that much for them! It is another system, not ours. Perhaps if I do it this way instead... That looks better, no, it is still too much. I said 500 when you ordered them, did I not? I will have to go another way. (Karin at Knutson February 2021)

While the mPOS interface at checkout remained unchanged, this example showed how the chain organisation’s new system of brand collaborations transformed the store into a marketplace. The stakeholders’ service systems intersected at the checkout, and the salesperson had to navigate different on-screen systems to complete the sale. As a result, the changes to the internal digital service ecosystem altered Karin’s role in the connected service encounter. However, it is only the inputs and outputs that are visible to the salesperson, while the internal workings of the expanded mPOS system are invisible. In salespeople’s service encounters, the ever-changing mPOS and service management systems are as a black box. Inside this box are features that can facilitate the checkout process, provide new services, and enhance salespeople’s role and agency the service encounter. Unpacking the box requires continuous learning and skills development. Borrowing Bowen’s (2016: 8) conceptualisation of four new FSE roles, the enabler, coordinator, innovator, and differentiator (or combinations thereof), two were identified in this research study. As an enabler, Karin helps both customers and technology to perform their respective roles in the service game, acting as a translator between the different languages of mPOS, customer, store, and retail organisation. Karin is also a coordinator, meaning she manages the interdependencies between the retail organisation’s and brand firms’ different service systems to create a successful outcome (cf. Bowen 2016; De Keyser et al. 2019; Evans and Kitchin 2018; Larivière et al. 2017). These findings indicate that the postdigital dialogue evolving in connected service encounters requires additional skills beyond the emotional labour and customer service that has hitherto characterised service work (e.g., Casaca 2012; Groth et al. 2019).

### Retail Chain Organisation’s Digitalisation Strategy and Covid-19

Covid-19 has changed how consumers purchase and providers sell services, groceries, shoes, clothes, home electronics, and other products (Faraj et al. 2021; Voorhees et al. 2020). In this research study, two opposite strategies for digitalising the service encounter in chain stores crystallised during the pandemic: *automated service encounters* and *servitised service encounters*. Here, automated refers to the substitution of salespeople with digital technologies in the service encounter. In servitised service encounters, digital technologies automate routine services and enhance salespeople’s role, skills, and performance. This finding concurs with several studies on the rapid digital transformation of frontline service and the service encounter before the pandemic (e.g., Brynjolfsson and McAfee 2011; Huang 2017; Ikeler 2016; Larivière et al. 2017).

Of the retail chain organisations surveyed, Bengtson was the furthest developed towards automated service encounters. The Bengtson store was connected to the online store through radio-frequency identification (RFID) technology, QR systems, and a smartphone application. Rather than asking a salesperson for help, the customer scanned the QR code on the item label to get further information about the product. During the observations, the salespeople were seen operating the checkout but rarely on the shopfloor, where they were occupied with replenishing and maintaining the displays rather than engaging with customers. On one occasion, a customer approached a salesperson for help but was directed to use the application on her smartphone. This finding shows that digital technologies, AI, and robots can indeed substitute FSEs in the service encounter (cf. Frude 2019; Sellar 2019).

In the late spring of 2021, self-checkouts based on QR systems and customers’ mobile devices were introduced in the Gustafson stores, which can be viewed as the first step towards automated service encounters (Sharma et al. 2021). Before the self-checkouts were introduced, concerns were raised among the employees about what would happen to their jobs and profession. As Gina voiced:

It is the personal thing I like, that is why I have stayed in this job so long. My work starts on the shopfloor, where I can help the customer to find products. We talk about different things, and I can give suggestions on products or makeup techniques. That is the best part of the job, the personal encounter. Then you follow the customer to the checkout to end the purchase. That is how we create excellent customer service and satisfied customers. Wouldn’t it be very impersonal if the customers had to checkout themselves? I cannot imagine what it would be like, not being part of the entire purchase process. What will happen with our jobs? (Gina at Gustafson May 2021)

However, the initial fear had lessened 4 months later, as it was apparent that the self-checkouts only concerned transactions and not customer service in the checkout practice. The customer still had to approach a salesperson to deactivate alarms and show the electronic receipt before leaving the store. Moreover, in August 2021, only a few customers had used the new self-checkout system.

A common feature of the retail organisations moving towards servitised service encounters was connected to their vision and mission statements, analytically theorised as social-political arrangements. These organisations emphasised service levels and employees’ role in creating service and customer experiences in the service encounter. The salespeople working in the servitised stores were aware of the company’s values and mission statements and embodied the service script. For instance, one of the retail organisations with a strong focus on personal service and digital FSE development predominantly employed salespeople with a background in hospitality. As one participant stated, ‘If you do not have experience as a hotel or spa receptionist, you are far down the list to get employment here’.

Anderson (USA) emerged from the data as the furthest developed retail organisation in terms of servitised connected service encounters. For example, in 2012, the company introduced MSAs; by 2015, MSAs were a standard tool in salespeople’s service encounters in all Anderson stores. An MSA can be used for mobile checkout, adding purchases, searching for items in the stock room, ordering items from the online store, demonstrating and comparing virtual products and brands, making skin analyses, or making virtual makeup on customers. As Anna put it:

It is really helpful with the iPad. I do not have to leave the customer to check for items at the checkout […] This spring, the company decided that all salespeople should use iPads in the service encounter to provide outstanding customer service to each customer. I think it is good because now you can follow the customer through the entire purchase process [and] give better service as the customer does not have to spend time queuing at the checkout to pay. (Anna at Anderson July 2015)

However, in the Swedish stores that had introduced MSAs, the devices were commonly kept at the fixed checkout, and the store managers emphasised that it was not a requirement to use them in the service encounter. In general, the participants were hesitant to adopt MSAs on the shopfloor and raised several concerns connected to understandings of the service encounter. For instance, one participant said:

I find it strange to use the iPad in the customer encounter. I think you lose the essence of the profession when you involve technology in the personal communication. I am here for the customers, and you need to be social to be a good salesperson. You must read the customer’s personality, and you cannot do that with an iPad. (Frida at Fredrikson May 2018)

Particularly revealing in the research was how the participants, no matter the age, connected the use of MSAs in the service encounter with rude behaviour. As one Bengtson salesperson put it:

Which salesperson would you go for, the one with a smartphone or the one without? I would go for the one without. (Beata at Bengtson August 2020)

During the pandemic, several retail chain organisations increased the number of MSAs in their stores, updated with additional applications to facilitate mobile checkouts as part of selling practices on the shopfloor. However, the MSAs were kept at the checkout and were only used occasionally on the shopfloor if customers asked for products that were not in stock. Furthermore, order taking emerged as a new task in salespeople’s service encounters. In their coordinator role, more time was spent on administrating customers’ online purchases and handling situations connected to online purchases. Increasingly, specific mobile devices –—‘zebras’ — were used for other tasks such as inventory. Nevertheless, it also emerged that many employees were either not interested or knowledgeable enough to adopt the new roles and tasks based on digital technologies.

A recent study of the impact of Covid-19 on the ‘forgotten frontline’ (retail, hospitality, personal service employees) in the USA reported that FSEs are facing a heightened amount of stress and uncertainty (Voorhees et al. 2020). However, in this research, neither the participants nor their colleagues had experienced increased stress or fear at work. However, they forwarded that physical restrictions — such as face masks, physical barriers, and social distancing — had constrained the interaction with customers. In conjunction with the easing of governmental restrictions (August 2021), many of the participants now thought differently about the digitalisation of service work. In the words of one participant working in one of the apparel stores:

Three years ago, there was talk that digitalisation would change our customer service and staffing. Now I do not believe in digitalisation anymore; it is just talking and not for real. Just look at how it is now. Customers want to meet real people when they enter the store, feel real clothes, and not touch a silly screen. They want personal service, and no technology can provide that. (Cecilia at Carlson August 2021)

While it triggered job losses and financial problems, the pandemic also reinforced the participants’ notion of the service encounter as *a game between people* in which the human senses are central. Their social respect and status increased as customers were appreciative of their engagement and knowledge. These findings must be seen in the light of Sweden responding differently to many other countries during the pandemic: retail stores and other service firms were never closed.

## Discussion

In the ongoing debate over the digitalisation of service work, researchers have argued that service jobs requiring emotional labour are less susceptible to automation. The untrainable attributes of social and emotional interactions are not easily programmed into the digital service and managerial infrastructure (Autor 2015; Frey and Osborne 2017; Frude 2019; Sellar 2019). However, other scholars provide evidence that emotional labour can follow a similar trajectory to that experienced in clerical and manufacturing work (e.g., Huang and Rust 2021; Ikeler 2016). This study provides additional evidence for both sides of the discussion. Two polarities of retail chain organisations’ digitalisation of service encounters were identified: *automated* and *servitised service encounter*s. In retail organisations developing towards automated service encounters, automating and thinking technologies (see Huang 2017) can substitute salespeople’s role in service encounters. When the creation of customer satisfaction and value creation is delegated to AI and robots, other tasks will substitute emotional labour or lead to technological unemployment when the traditional store becomes obsolete. In contrast, organisations developing towards servitised service encounters open opportunities for enhancing (rather than replacing) service work. Thinking technologies have the potential to boost FSEs’ actions and interactions in the service encounter, although this will require learning how best to collaborate with AI technology in various situations (see De Keyser et al. 2019).

**Fig. 4:**
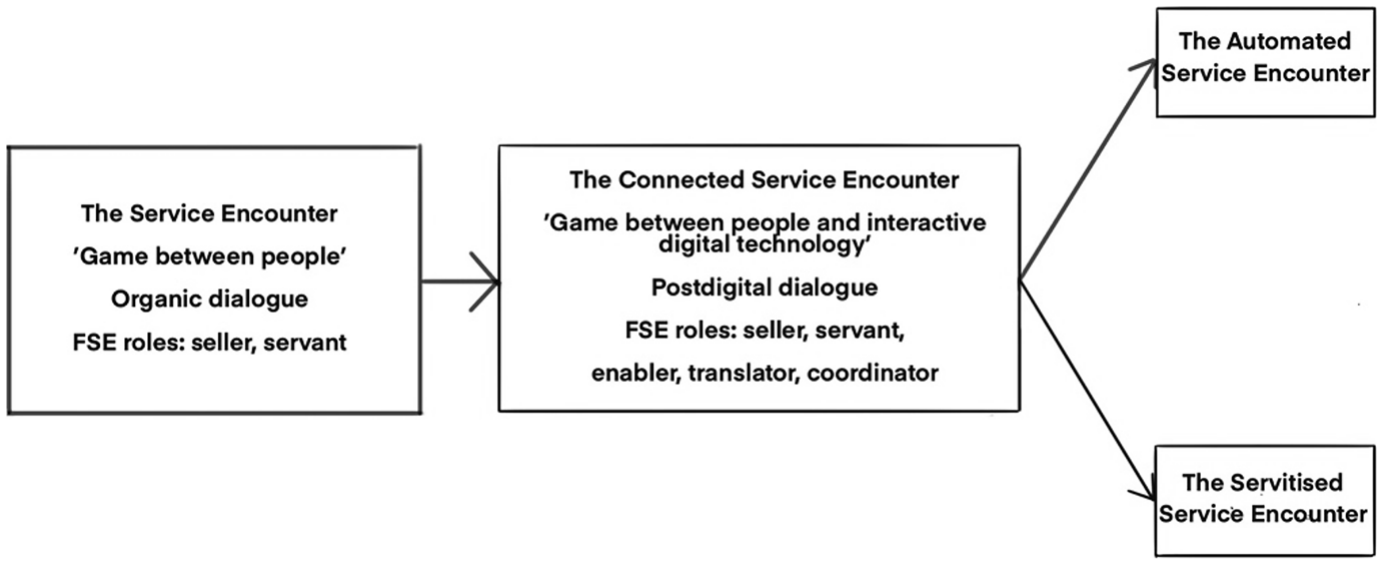
The evolution of FSEs’ service encounters, from organic to postdigital dialogue

However, it is a significant leap to go from using an mPOS system as a tool for transactions in the checkout practice to collaborating with AI and robot technologies in service encounters. The salespeople interviewed could not imagine how such technology could be used in their work. As with teachers’ concerns about implementing such technologies in classroom teaching (Hrastinski et al. 2019), they expressed concerns about being replaced by a robot. The participants also dismissed the idea of such a future happening, arguing that digital technology could never substitute their work as emotional labour. A plausible explanation for this statement is that a large proportion of the retail organisations in the research had developed from connected service encounters towards servitised service encounters (Fig. 4); the digitalisation of frontline service in these workplaces brought new tasks and job positions that required FSEs to engage in workplace learning. Whether service organisations target automated or servitised service encounters, the future for FSEs with low literacy, numeracy, computer, and digital skills is precarious. It is also important to point out that connected service encounters (based on interactive digital service networks) are not yet widespread. Ultimately, if they lack the resources to adapt to rapid changes in the service organisation and adopt new service roles, FSEs in entry-level positions (e.g., retail, hospitality, customer service) will be marginalised in the connected and servitised service encounter (cf. Barocas and Levy 2016; Ikeler 2016; Nickson et al. 2012).

The postdigital seeks to understand how humans and technology relate to one another beyond binaries: ‘we are increasingly no longer in a world where digital technology and media is separate, virtual other to a natural human and social life’(Jandrić et al. 2018). The findings in this study show that the practice traditions that reproduce salespeople’s service encounters over time are resistant to change. Despite the retail chain organisations’ efforts to implement in-situ technologies intended for use by customers and salespeople on the shopfloor, the salespeople reproduced service encounters defined as a game between people (Solomon et al. 1985), characterised by organic dialogue and emotional labour skills. At checkout, the service encounter transformed into what I define as a game between people and interactive technology, a connected service encounter characterised by ‘postdigital dialogue’ (Jandrić et al. 2019). The checkout process involved two parts, namely transactions and customer service. Three roles connected to transactions were identified: the enabler, translator, and coordinator (see Bowen 2016). However, the specifics of service work in the connected service encounter are not that emotional labour associated with customer service is expanded with numeracy, literacy, economic, computer, and digital skills connected to transactions. In the postdigital dialogue, the FSE interacts with the mPOS system and the customer simultaneously. Furthermore, as mPOS system interfaces are largely unchanged compared to older POS systems, while the constantly evolving mPOS software and service ecosystems are black boxed, there is a significant risk that the changes in skills requirements will remain hidden.

Across the literature on emotional labour, conceptualisations of skills relate to the service encounter as a game between the service worker and customer. Among others, Subramony et al. (2021), Voorhees et al. (2020), Schneider and Bowen (2019), and Larivière et al. (2017) emphasise the need for more extensive research on FSEs’ attitudes, behaviours, and acceptance of new roles. However, this article argues that coming to practice service encounters differently involves changing or replacing a combination of cultural-discursive arrangements (ideas thought about the service encounter, digital technologies, the wider profession), material-economic arrangements (store layout, furnishings, routines, methods, employee development), and social-political arrangements (service script, values, relationships) that keep present service encounters stable. Service research has long considered how store layout, furnishings, light, and music impact interactions in the service encounter (e.g., Bitner 1992). However, the knowledge of how digital conditions transform FSEs’ work and well-being remains limited.

## Conclusions

This article outlines an ethnography of salespeople’s service encounters in connected speciality stores between July 2015 and August 2021. Its contributions to the ongoing debate over the digitalisation of service work are twofold. On the theoretical plane, the article provides an alternative framework to labour process theory, namely the theory of practice architectures, as a means to explore and describe the digitalisation of service work from a postdigital perspective. A conceptual diagram is developed to visualise the complex web of conditions that form the practice architectures of salespeople’s selling practices as part of the connected service encounter. This article’s second contribution uncovers those conditions that change salespeople’s practice of service encounters and those attributes associated with service work and emotional labour skills. The research has shown that the connected service encounter is characterised by ‘postdigital dialogue’ (Jandrić et al. 2019: 164) and involves new FSE roles and skills, arguing that the transformation required of FSEs in postdigital service practices will be produced by FSEs changing not just their emotions, feelings, values, and relationships but also their practice of service encounters, and by people (employer, managers, co-workers, customers) constructing new conditions to support those changed practices. Together, the findings can contribute to a better understanding of how digitalisation changes FSEs’ actions and interactions in service encounters.

A limitation of this study is that the sample was restricted to service encounters in speciality stores. A broader sample — including, for example, supermarkets and department and outlet stores — may have elicited further aspects of the digitalisation of service work. Notwithstanding this limitation, the study suggests that future research should take an interdisciplinary approach to service work and learning. Further research should be undertaken to explore how workplace learning, lifelong learning, and formal education address the digitalisation of frontline service work and the service encounter.

### Declarations

The study reports on a longitudinal study conducted through the ethnographic methods of participant observation (covert, overt, shadowing), interviews, and field interviews. All procedures followed were in accordance with the ethical standards in international guidelines and the ethical guidelines of the Swedish Ethical Review Act (SFS 2003:460). The research was based on the intention of contributing to the development of new knowledge through ethnographic research methods, and that the well-being of the participants should be given priority ahead of the needs of society and science. Because the study was designed to detect the realities of service employees’ work that are largely unknown to the public, using covert and overt participant observation was well justified. First, since the study intended to record physical service encounter settings without identifying the people I observed and interacted with or their workplaces, this ethnographic work would pose insignificant risks to its anonymous participants. All retail organisations, workplaces, and participants were assigned pseudonyms in the field notes. Second, the data production took place in constantly changing public social settings, with a flow of new customers and situations. The disclosure of my research identity when conducting covert participant observations was neither necessary nor practical; doing so would interrupt the salespeople’s routines of service work. In overt participant observations and field interviews, I revealed my research identity, described the purpose of the research and how the data would be handled. The process of gaining informed consent was ongoing throughout the research engagement and was renegotiated on each occasion. No personal information was recorded, and the field notes were written afterwards outside the workplace.

## References

[CR1] Autio, E., Mudambi, R., & Yoo, Y. (2021). Digitalization and globalization in a turbulent world: Centrifugal and centripetal forces. *Global Strategy Journal*, *11*(1), 3-16. 10.1002/gsj.1396.

[CR2] Autor, D. H. (2015). Why are there still so many jobs? The history and future of workplace automation. *Journal of Economic Perspectives*, *29*(3), 3-30. 10.1257/jep.29.3.3.

[CR3] Barocas, S., & Levy, K. (2016). What customer data collection could mean for workers. Harvard Business Review, 31 August. https://hbr.org/2016/08/the-unintended-consequence-of-customer-data-collection. Accessed 14 December 2021.

[CR4] Basker, E. (2016). The evolution of technology in the retail sector. In E. Basker (Ed.), *Handbook on the Economics of Retailing and Distribution* (pp. 38–53). Edward Elgar Publishing Limited. 10.4337/9781783477388.00010.

[CR5] Bell, D. (1973). *The coming of post-industrial society: a venture in social forecasting*. New York: Basic books.

[CR6] Bitner, M. J. (1992). Servicescapes: The impact of physical surroundings on customers and employees. *Journal of Marketing*, *56*(2), 57-71. 10.1177/2F002224299205600205.

[CR7] Bolton, S. C. (2004). *Emotion management in the workplace*. Basingstoke: Macmillan.

[CR8] Bowen, D. E. (2016). The changing role of employees in service theory and practice: An interdisciplinary view. *Human resource management review*, *26*(1), 4-13. 10.1016/j.hrmr.2015.09.002.

[CR9] Brynjolfsson, E., Hu, Y. J., & Rahman, M. (2013). Competing in the Age of Omnichannel Retailing. *MIT Sloan Management Review*, *54*(4), 23-29.

[CR10] Brynjolfsson, E., & McAfee, A. (2011). *Race against the machine: how the digital revolution is accelerating innovation, driving productivity, and irreversibly transforming employment and the economy*. Lexington: Digital Frontier.

[CR11] Bélanger, J., & Edwards, P. (2013). The nature of front-line service work: distinctive features and continuity in the employment relationship. *Work, Employment and Society, 27*(3), 433-450. 10.1177/0950017013481877.

[CR12] Casaca, S. F. (2012). Behind smiles and pleasantness: working in the interactive service sector in Portugal. *International Journal of Work Organisation and Emotion*, *5*(1), 56. 10.1504/IJWOE.2012.048592.

[CR13] Czarniawska, B. (2007). *Shadowing and other techniques for doing fieldwork in modern societies*. Malmö/Copenhagen/Oslo: Liber/CBS Press/Universitetsforlaget.

[CR14] De Keyser, A., Köcher, S., Alkire, L., Verbeeck, C., & Kandampully, J. (2019). Frontline Service Technology infusion: conceptual archetypes and future research directions. *Journal of Service Management*, *30*(1), 156-183. 10.1108/JOSM-03-2018-0082.

[CR15] Evans, L., & Kitchin, R. (2018). A smart place to work? Big data systems, labour, control and modern retail stores. *New Technology, Work and Employment*, *33*(1), 44-57. 10.1111/ntwe.12107.

[CR16] Faraj, S., Renno, W., & Bhardwaj, A. (2021). Unto the breach: What the COVID-19 pandemic exposes about digitalization. *Information and Organization, 31*(1), 100337. 10.1016/j.infoandorg.2021.100337.

[CR17] Frey, C. B., & Osborne, M. A. (2017). The future of employment: How susceptible are jobs to computerisation? *Technological Forecasting & Social Change*, *114*(C), 254–280. 10.1016/j.techfore.2016.08.019.

[CR18] Frude, N. (2019). Technological Unemployment and Psychological Well-being—Curse or Benefit? In M. A. Peters, P. Jandrić, & A. Means (Eds.), *Education and technological unemployment* (pp. 95–113). Singapore: Springer. 10.1007/978-981-13-6225-5_7.

[CR19] Groth, M., Wu, Y., Nguyen, H., & Johnson, A. (2019). The moment of truth: A review, synthesis, and research agenda for the customer service experience. *Annual Review of Organizational Psychology and Organizational Behavior, 6, *89–113. 10.1146/annurev-orgpsych-012218-015056.

[CR20] Hagberg, J., Jonsson, A., & Egels-Zandén, N. (2017). Retail digitalization: Implications for physical stores. *Journal of Retailing and Consumer Services*, *39*, 264-269. 10.1016/J.JRETCONSER.2017.08.005.

[CR21] Hastwell, K., Strauss, P., & Kell, C. (2013). ‘But pasta is pasta, it is all the same’: The language, literacy and numeracy challenges of supermarket work. *Journal of Education and Work*, *26*(1), 77-98. 10.1080/13639080.2011.629181.

[CR22] Hochschild, A. R. (1983). *The managed heart: commercialization of human feeling*. Berkeley, CA: University of California Press.

[CR23] Howcroft, D., & Rubery, J. (2019). 'Bias in, Bias out': gender equality and the future of work debate. *Labour & industry (Brisbane, Qld.)*, *29*(2), 213–227. 10.1080/10301763.2019.1619986.

[CR24] Hrastinski, S., Arkenback-Sundström, C., D. Olofsson, A., Ekström, S., Ericsson, E., Fransson, G., . . . Utterberg, M. (2019). Critical Imaginaries and Reflections on Artificial Intelligence and Robots in Postdigital K-12 Education. *Postdigital Science and Education*, *1*(2), 427-445. 10.1007/s42438-019-00046-x.

[CR25] Huang, M.-H. (2017). Technology in the Frontline: From Dumb to Thinking to Feeling In A. Rafaeli, D. Altman, D. D. Gremler, M.-H. Huang, D. Grewal, B. Iyers, A. Parasuraman, & K. d. Ruyter (Eds.), *The Future of Frontline Research: Invited Commentaries (Journal of Service Research, 29, *pp. 93–95). 10.1177/1094670516679275.

[CR26] Huang, M.-H., & Rust, R. T. (2021). Engaged to a robot? The role of AI in service. * Journal of Service Research, 24*(1), 30-41. 10.1177/1094670520902266.

[CR27] Ikeler, P. (2016). Deskilling emotional labour: Evidence from department store retail. *Work, Employment and Society, 30*(6), 966-983. 10.1177/0950017015609031.

[CR28] Ilsøe, A. (2017). The digitalisation of service work – social partner responses in Denmark, Sweden and Germany. *Transfer: European Review of Labour and Research*, *23*(3), 333-348. 10.1177/1024258917702274.

[CR29] Jandrić, P., Knox, J., Besley, T., Ryberg, T., Suoranta, J., & Hayes, S. (2018). Postdigital science and education. *Educational philosophy and theory*, *50*(10), 893-899. 10.1080/00131857.2018.1454000.

[CR30] Jandrić, P., Ryberg, T., Knox, J., Lacković, N., Hayes, S., Suoranta, J., . . . Gibbons, A. (2019). Postdigital Dialogue. *Postdigital Science and Education, 1*(1), 163–189. 10.1007/s42438-018-0011-x.

[CR31] Kemmis, S. (2019). *A Practice Sensibility: An Invitation to the Theory of Practice Architectures*. Singapore: Springer.

[CR32] Kemmis, S., Edwards-Groves, C., Jakhelln, R., Choy, S., Wärvik, G.-B., Gyllander Torkildsen, L., & Arkenback-Sundström, C. (2020). Teaching as Pedagogical Praxis. In K. Mahon, C. Edwards-Groves, S. Fransisco, M. Kaukko, S. Kemmis, & K. Petrie (Eds.), *Pedagogy, Education, and Praxis in Critical Times* (pp. 85–116). Singapore: Springer. 10.1007/978-981-15-6926-5_5.

[CR33] Kemmis, S., & Grootenboer, P. (2008). Situating praxis in practice: Practice architectures and the cultural, social and material conditions for practice. In P. Salo & S. Kemmis (Eds.), *Enabling Praxis: Challenges for education* (pp. 37–64). Rotterdam: Sense. 10.1163/9789087903275_004.

[CR34] Kemmis, S., Wilkinson, J., Edwards-Groves, C., Hardy, I., Grootenboer, P., & Bristol, L. (2014). *Changing practices, changing education*. Singapore: Springer.

[CR35] Knox, J. (2019). What Does the ‘Postdigital’ Mean for Education? Three Critical Perspectives on the Digital, with Implications for Educational Research and Practice. *Postdigital Science and Education, 1*(2), 357-370. 10.1007/s42438-019-00045-y.

[CR36] Korczynski, M. (2002). *Human resource management in service work*. Basingstoke: Palgrave.

[CR37] Korczynski, M. (2005). Skills in service work: an overview. *Human Resource Management Journal*, *15*(2), 3-14. 10.1111/j.1748-8583.2005.tb00143.x.

[CR38] Larivière, B., Bowen, D., Andreassen, T. W., Kunz, W., Sirianni, N. J., Voss, C., . . . De Keyser, A. (2017). “Service Encounter 2.0”: An investigation into the roles of technology, employees and customers. *Journal of business research*, *79*, 238-246. 10.1016/j.jbusres.2017.03.008.

[CR39] Leidner, R. (1993). *Fast food, fast talk: Service work and the routinization of everyday life*. Berkeley, CA: University of California Press.

[CR40] Mahon, K., Edwards-Groves, C., Francisco, S., Kaukko, M., Kemmis, S., & Petrie, K. (2020). *Pedagogy, Education, and Praxis in Critical Times*. Singapore: Springer.

[CR41] Mahon, K., Francisco, S., & Kemmis, S. (2017a). *Exploring Education and Professional Practice: Through the Lens of Practice Architectures*. Singapore: Springer. 10.1007/978-981-10-2219-7.

[CR42] Mahon, K., Francisco, S., Kemmis, S., & Lloyd, A. (2017b). Introduction: Practice theory and the theory of practice architectures. In K. Mahon, S. Fransisco, & S. Kemmis (Eds.), *Exploring Education And Professional Practice – Through The Lens Of Practice Architectures *(pp. 1-30). Singapore: Springer. 10.1007/978-981-10-2219-7_1.

[CR43] McAfee, A., & Brynjolfsson, E. (2014). *The second machine age: work, progress, and prosperity in a time of brilliant technologies*. New York: W. W. Norton & Company.

[CR44] Moore, S., & Hayes, L. J. B. (2017). Taking worker productivity to a new level? Electronic Monitoring in homecare—the (re)production of unpaid labour. *New Technology, Work and Employment*, *32*(2), 101-114. 10.1111/ntwe.12087.

[CR45] Nickson, D., Warhurst, C., Commander, J., Hurrell, S. A., & Cullen, A. M. (2012). Soft skills and employability: Evidence from UK retail. *Economic and Industrial Democracy*, *33*(1), 65-84. 10.1177/0143831X11427589.

[CR46] Nickson, D., Warhurst, C., Cullen, A. M., & Watt, A. (2003). Bringing in the Excluded? Aesthetic labour, skills and training in the 'new' economy. *Journal of Education and Work*, *16*(2), 185-203. 10.1080/13639080305560.

[CR47] O'Reilly, K. (2012). *Ethnographic methods*. Abingdon: Routledge.

[CR48] Payne, J. (2009). Emotional Labour and Skill: A Reappraisal. *Gender, Work & Organization*, *16*(3), 348-367. 10.1111/j.1468-0432.2009.00448.x.

[CR49] Rafaeli, A., Altman, D., Gremler, D. D., Huang, M.-H., Grewal, D., Iyer, B., de Ruyter, K. (2017). The future of frontline research: invited commentaries. *Journal of Service Research, 20*(1), 91–99. 10.1177/2F1094670516679275.

[CR50] Schneider, B., & Bowen, D. E. (2019). Perspectives on the Organizational Context of Frontlines: A Commentary. *Journal of Service Research, 22*(1), 3–7. 10.1177/2F1094670518816160.

[CR51] Sellar, S. (2019). Acceleration, Automation and Pedagogy: How the Prospect of Technological Unemployment Creates New Conditions for Educational Thought. In M. Peters, P. Jandrić, & A. Means (Eds.), *Education and Technological Unemployment* (pp. 131–144). Singapore: Springer Singapore. 10.1007/978-981-13-6225-5_9.

[CR52] Sharma, P., Ueno, A., & Kingshott, R. (2021). Self-service technology in supermarkets – Do frontline staff still matter? *Journal of Retailing and Consumer Services, 59*, 102356. 10.1016/j.jretconser.2020.102356.

[CR53] Shove, E., Pantzar, M., & Watson, M. (2012). *The dynamics of social practice: Everyday life and how it changes*. Sage.

[CR54] Singh, J., Brady, M., Arnold, T., & Brown, T. (2017). The Emergent Field of Organizational Frontlines. *Journal of service research: JSR*, *20*(1), 3–11. 10.1177/2F1094670516681513.

[CR55] Solomon, M. R., Surprenant, C., Czepiel, J. A., & Gutman, E. G. (1985). A role theory perspective on dyadic interactions: the service encounter. *Journal of Marketing, 49*(1), 99-111. 10.2307/1251180.

[CR56] Spencer, D. A. (2018). Fear and hope in an age of mass automation: debating the future of work. *New Technology, Work and Employment*, *33*(1), 1-12. 10.1111/ntwe.12105.

[CR57] Spreer, P., & Rauschnabel, P. A. (2016). Selling with technology: understanding the resistance to mobile sales assistant use in retailing. *Journal of Personal Selling & Sales Management*, *36*(3), 240-263. 10.1080/08853134.2016.1208100.

[CR58] Subramony, M., Groth, M., Hu, X. J., & Wu, Y. (2021). Four Decades of Frontline Service Employee Research: An Integrative Bibliometric Review. *Journal of Service Research*, *24*(2), 230-248. 10.1177/1094670521999721.

[CR59] Surprenant, C. F., & Solomon, M. R. (1987). Predictability and Personalization in the Service Encounter. *Journal of Marketing*, *51*(2), 86-96. 10.1177/002224298705100207.

[CR60] Tolich, M., & Briar, C. (1999). Just Checking It Out: Exploring the Significance of Informal Gender Divisions Amongst American Supermarket Employees. *Gender, Work & Organization*, *6*(3), 129-133. 10.1111/1468-0432.00076.

[CR61] Verhoef, P. C., Kannan, P. K., & Inman, J. J. (2015). From Multi-Channel Retailing to Omni-Channel Retailing: Introduction to the Special Issue on Multi-Channel Retailing: Introduction to the Special Issue on Multi-Channel Retailing. *Journal of Retailing*, *91*(2), 174–181. 10.1016/j.jretai.2015.02.005.

[CR62] Voorhees, C. M., Fombelle, P. W., & Bone, S. A. (2020). Don’t forget about the frontline employee during the COVID-19 pandemic: Preliminary insights and a research agenda on market shocks. *Journal of Service Research*. 10.1177/2F1094670520944606.

[CR63] Warhurst, C., Van den Broek, D., Hall, R., & Nickson, D. (2012). Great expectations: gender, looks and lookism at work. *International Journal of Work Organisation and Emotion*, *5*(1), 72. 10.1504/IJWOE.2012.048593.

[CR64] Webb, L. (2017). Online Research Methods, Qualitative. In J. Matthes (Ed.), *The International Encyclopedia of Communication Research Methods*. Hoboken, NJ: Wiley. 10.1002/9781118901731.iecrm0173.

[CR65] Webb, S. (2012). Online tutoring and emotional labour in the private sector. *Journal of Workplace Learning*, *24*(5), 365-388. 10.1108/13665621211239895.

[CR66] Webster, J. (2004). Digitising inequality: the cul-de-sac of women's work in European services. *New Technology, Work and Employment*, *19*(3), 160-176. 10.1111/j.1468-005X.2004.00135.x.

[CR67] Wharton, A. (2009). The Sociology of Emotional Labor. *Annual Review of Sociology, 35, *147-165. 10.1146/annurev-soc-070308-115944.

[CR68] Wilson, R. (2019). Skills forecasts in a rapidly changing world: Through a glass darkly. In S. McGrath, M. Mulder, J. Papier, & R. Suart (Eds.), *Handbook of Vocational Education and Training Developments in the Changing World of Work*. Singapore: Springer. 10.1007/978-3-319-94532-3.

[CR69] Yrjölä, M., Spence, M. T., & Saarijärvi, H. (2018). Omni-channel retailing: propositions, examples and solutions. *The International Review of Retail, Distribution and Consumer Research*, *28*(3), 259-276. 10.1080/09593969.2018.1445657.

